# In vitro evaluation of (*S*)-2-amino-3-[3-(2-^18^F-fluoroethoxy)-4-iodophenyl]-2-methylpropanoic acid (^18^F-FIMP) as a positron emission tomography probe for imaging amino acid transporters

**DOI:** 10.1186/s13550-023-00988-1

**Published:** 2023-04-28

**Authors:** Satoshi Nozaki, Yuka Nakatani, Aya Mawatari, William Ewan Hume, Hisashi Doi, Yasuyoshi Watanabe

**Affiliations:** 1 Laboratory for Pathophysiological and Health Science, RIKEN Center for Biosystems Dynamics Research, 6-7-3 Minatojima-Minamimachiinamimachi, Chuo-Ku, Kobe, Hyogo 650-0047 Japan; 2 Novel PET Diagnostics Laboratory, RIKEN Innovation Center, Hyogo, Japan; 3 Laboratory for Labeling Chemistry, RIKEN Center for Biosystems Dynamics Research, Kobe, Hyogo Japan

**Keywords:** Tumor, Amino acid transporter, L-type amino acid transporter 1, Positron emission tomography

## Abstract

**Background:**

(*S*)-2-amino-3-[3-(2-^18^F-fluoroethoxy)-4-iodophenyl]-2-methylpropanoic acid (^18^F-FIMP) as a promising PET probe for imaging the tumor-specific L-type amino acid transporter (LAT) 1. Our previous study revealed that ^18^F-FIMP had a higher affinity for LAT1 than for LAT2 abundantly expressed even in normal cells. ^18^F-FIMP showed high accumulation in LAT1-positive tumor tissues and low accumulation in inflamed lesions in tumor-bearing mice. However, the affinity of ^18^F-FIMP for other amino acid transporters was not determined yet. Here, we aimed to determine whether ^18^F-FIMP has affinity for other tumor-related amino acid transporters, such as sodium- and chloride-dependent neutral and basic amino acid transporter B(0 +) (ATB^0,+^), alanine serine cysteine transporter 2 (ASCT2), and cystine/glutamate transporter (xCT).

**Procedures:**

Cells overexpressing LAT1, ATB^0,+^, ASCT2, or xCT were established by the transfection of expression vectors for LAT1, ATB^0,+^, ASCT2, or xCT. Protein expression levels were determined by western blot and immunofluorescent analyses. Transport function was evaluated by a cell-based uptake assay using ^18^F-FIMP and ^14^C-labeled amino acids as substrates.

**Results:**

Intense signals were observed only for expression vector-transfected cells on western blot and immunofluorescent analyses. These signals were strongly reduced by gene-specific small interfering ribonucleic acid treatment. The uptake values for each ^14^C-labeled substrate were significantly higher in the transfected cells than in the mock-transfected cells and were significantly inhibited by the corresponding specific inhibitors. The ^18^F-FIMP uptake values were significantly higher in the LAT1- and ATB^0,+^-overexpressing cells than in the corresponding mock cells, but no such increase was seen in the ASCT2- or xCT-overexpressing cells. These ^18^F-FIMP uptake values were significantly decreased by the specific inhibitors for LAT1- and ATB^0,+^.

**Conclusions:**

We demonstrated that ^18^F-FIMP has affinity not only for LAT1, but also for ATB^0,+^. Our results may be helpful for understanding the mechanisms of the whole-body distribution and tumor accumulation of ^18^F-FIMP.

**Supplementary Information:**

The online version contains supplementary material available at 10.1186/s13550-023-00988-1.

## Background

Positron emission tomography (PET) imaging can assist in early phase clinical evaluations of tumors. 2-Deoxy-2-^18^F-fluoro-D-glucose (FDG) is the most commonly used PET probe for tumor imaging as it has high uptake in tumor cells, which are known to have a high glucose requirement. However, ^18^F-FDG is actively transported into cells via glucose transporters, which are enriched not only in tumor tissues, but also in inflamed lesions, where they regulate glucose metabolism to facilitate the inflammatory response [1]. Hence, ^18^F-FDG PET cannot be used to distinguish between tumor tissues and inflamed lesions [2], and may therefore result in false positives for tumor diagnosis [3].

Amino acids are essential nutrients required for the growth and survival of all types of cells. Tumor cells require high amounts of amino acids due to their high proliferative rate. All amino acids are hydrophilic and cannot pass through the cell membrane without specific amino acid transporters. Many amino acid transporters have already been identified, and four types of amino acid transporters, L-type amino acid transporter 1 (LAT1, also known as SLC7A5) [4], sodium- and chloride-dependent neutral and basic amino acid transporter B(0 +) (ATB^0,+^, also known as SLC6A14) [5], alanine serine cysteine transporter 2 (ASCT2, also known as SLC1A5) [6], and cystine/glutamate transporter (xCT, also known as SLC7A11) [7], are reportedly highly expressed in tumor cells (Table 1). These transporters have been shown to be involved in cell proliferation, apoptosis, epigenesis, reduction of oxidative stress, and drug resistance via cellular signaling pathways, such as mammalian target of rapamycin signaling [8–11].

**Table 1:** Properties of tumor-related amino acid transporters

Gene	Alias	Substrates	Inhibitors	PET probes
*SLC7A5*	*LAT1*	Leu, Ile, Val, Trp, Tyr, Phe, Met, His	BCH, JPH203	FAMT, FBPA, FACBC, FET, FPhPA
*SLC6A14*	*ATB*^*0,*+^	Leu, Ile, Val, Trp, Tyr, Phe, Met, His, Ala, Ser, Cys	α-Methyl-L-Trp	FEMAET, FET
*SLC1A5*	*ASCT2*	Ala, Ser, Cys, Thr, Gln, Asn	γ-Glu-p-nitroanilide, Benzylserine	FACBC, FPhPA
*SLC7A11*	*xCT*	Cys, Glu	Sulfasalazine, Erastin, Sorafenib	FSPG

PET, positron emission tomography; LAT1, L-type amino acid transporter 1; ATB^0,+^, sodium- and chloride-dependent neutral and basic amino acid transporter B(0 +); ASCT2, alanine serine cysteine transporter 2; xCT, cystine/glutamate transporter; BCH, 2-amino-2-norbornanecarboxylic acid; JPH203, (*S*)-2-amino-3-(4-((5-amino-2-phenylbenzo[d]oxazol-7-yl)methoxy)-3,5-dichloropheyl) propanoic acid; FAMT, L-3-^18^F-fluoro-α-methyl tyrosine; FBPA, 4-borono-2-^18^F-fluoro-phenylalanine; FACBC, anti-1-amino-3-^18^F-fluorocyclobutane-1-carboxylic acid; FET, ^18^F-fluoro-ethyl-tyrosine; FPhPA, 2-amino-5-(4-^18^F-fluorophenyl)pent-4-ynoic acid; FEMAET, *O*-2(2-^18^F-fluoroethyl)methylamino)ethyltyrosine; FSPG, (*4S*)-4-(3-^18^F-fluoropropyl)-L-glutamate

LAT1 is one of the sodium-independent L-type amino acid transporters [12]. LAT1 is highly expressed in various human tumors and is therefore a promising target for both imaging and therapeutics [13]. Several PET probes targeting LAT1 have been reported, including ^18^F-fluoro-ethyl-tyrosine (FET) [14] and L-3-^18^F-fluoro-α-methyl-tyrosine (FAMT) [15]. However, none of them has been widely applied that prevents the detection of significant differences. Recently, PET imaging using ^18^F-FACBC (^18^F-fluciclovine) has been developed and may solve various problems of PET imaging targeting amino acid transporters such as distinguishing between tumor and normal tissue in brain tumors, and determining the degree of malignancy [16–19].

We recently developed (*S*)-2-amino-3-[3-(2-^18^F-fluoroethoxy)-4-iodophenyl]-2-methylpropanoic acid (^18^F-FIMP) as a promising PET probe for imaging the tumor-specific amino acid transporter LAT1. ^18^F-FIMP showed high accumulation in LAT1-positive tumor tissues and low accumulation in inflamed tissues in tumor-bearing mice [20]. Tumor uptake of ^18^F-FIMP, but neither of ^11^C-methionine (MET) nor ^18^F-FDG, was effectively decreased 1 day after irradiation in LAT1-positive tumor-bearing mice [21]. Moreover, in comparison with ^11^C-MET and ^18^F-FDG, ^18^F-FIMP PET resulted in extremely clear images in patients with suspected glioblastoma [22]. ^18^F-FIMP was screened by a cell-based assay using cell lines that overexpressed LAT1 or LAT2. However, the affinity of ^18^F-FIMP for other amino acid transporters remains unknown, and it is unknown whether only LAT1 is involved in the accumulation mechanism of ^18^F-FIMP in tumor tissues. Here, we aimed to determine whether ^18^F-FIMP has affinity for other tumor-related amino acid transporters.

## Methods

### Cell cultures

CHO-K1 (RCB0285) Chinese hamster ovary cells, T3M-4 (RCB1021) human pancreatic adenocarcinoma cells, A549 (RCB0098) human lung carcinoma cells, and MCF7 (RCB1904) human breast adenocarcinoma cells were obtained from the RIKEN BioResource Research Center through the National Bio-Resource Project of the Ministry of Education, Culture, Sports, Science and Technology (MEXT) and the Japan Agency for Medical Research and Development (AMED), Japan. CHO-K1 and T3M-4 cells were cultured in Ham’s F12 medium (Nacalai Tesque, Inc., Kyoto, Japan) supplemented with 10% fetal bovine serum (FBS; Equitech-Bio, Inc., Kerrville, TX), 100 units/mL penicillin, and 100 μg/mL streptomycin (Nacalai Tesque, Inc.). A549 cells were cultured in Dulbecco’s Modified Eagle’s Medium (DMEM; Nacalai Tesque, Inc.) supplemented with 10% FBS (Equitech-Bio, Inc.), 100 units/mL penicillin, and 100 μg/mL streptomycin (Nacalai Tesque, Inc.). MCF7 cells were cultured in Minimum Essential Medium (MEM; Nacalai Tesque, Inc.) supplemented with 10% FBS (Equitech-Bio, Inc.), 1 mM sodium pyruvate, 0.1 mM nonessential amino acids, 100 units/mL penicillin, and 100 μg/mL streptomycin (Nacalai Tesque, Inc.). NCI-H460 (ATCC HTB-177) human lung cancer cells were obtained from the American Type Culture Collection, Manassas, VA. NCI-H460 cells were cultured in Roswell Park Memorial Institute 1640 medium (Nacalai Tesque, Inc.) supplemented with 10% FBS (Equitech-Bio, Inc.), 100 units/mL penicillin, and 100 μg/mL streptomycin (Nacalai Tesque, Inc.).

### Establishment of stably transfected cell lines

The coding regions of *LAT1* (*SLC7A5*; GenBank accession no. NM_003486, nucleotides 78 – 1601), *ATB*^*0,*+^ (*SLC6A14*; GenBank accession no. NM_007231, nucleotides 132 – 2060), and *xCT* (*SLC7A11*; GenBank accession no. NM_014331, nucleotides 281 – 1786) were amplified by polymerase chain reaction (PCR) with the introduction of *Sal*I and *Not*I sites, then ligated into the *Sal*I and *Not*I sites of the pGEM-T Easy Vector system (Promega, Madison, WI). The coding region of *ASCT2* (*SLC1A5*; GenBank accession no. NM_005628, nucleotides 621 – 2246) was amplified by PCR with the introduction of *Eco*R V and *Not*I sites, then ligated into the *Eco*R V and *Not*I sites of the pGEM-T Easy Vector system (Promega). These inserts were subcloned into the pEBMulti-Hyg vector (Fujifilm Wako Pure Chemical Corporation, Tokyo, Japan). These constructs were referred to as pEBMulti-Hyg-LAT1, pEBMulti-Hyg-ATB^0,+^, pEBMulti-Hyg-xCT, and pEBMulti-Hyg-ASCT2, respectively.

For the transfection of these expression vectors, CHO-K1 cells were seeded onto a 100-mm dish at a density of 1 × 10^6^ to 2 × 10^6^ cells/dish and cultured overnight. At 60% to 70% confluence, the cells were transfected with 10 μg of the pEBMulti-Hyg vector (mock) or pEBMulti-Hyg-LAT1 by using TransIT-X2 (Mirus Bio LLC, Madison, WI), pEBMulti-Hyg-ATB^0,+^ or pEBMulti-Hyg-ASCT2 by using TurboFectin 8.0 (OriGene Technologies, Inc., Rockville, MD), or pEBMulti-Hyg-xCT by using ViaFect (Promega) according to the manufacturers’ protocols. Subsequently, at 24 to 48 h after transfection, cells were subcultured into a 100-mm dish containing culture medium supplemented with Hygromycin B Gold (0.4 mg/mL; InvivoGen, San Diego, CA) for 2 to 3 weeks. The cells were then seeded onto a 96-well plate with a single cell per well. The culture medium was changed every 2 days for 2–3 weeks until a single colony could be seen. Cells were trypsinized, then transferred into a 24-well plate containing 500 μL of culture medium supplemented with Hygromycin B Gold. The cells were subcultured into a 6-well plate, and subsequently into a 100-mm dish, and maintained in the presence of Hygromycin B Gold.

For the transfection of gene-specific small interfering ribonucleic acids (siRNAs), each of the overexpressing cell lines were seeded onto a 100-mm dish at a density of 2 × 10^6^ cells/dish and cultured overnight. At 60–70% confluence, cells were transfected with 50 to 100 nM of gene-specific siGENOME human siRNA (Dharmacon, Inc., Lafayette, CO) by using Lipofectamine 3000 Reagent (Thermo Fisher Scientific, Waltham, MA) according to the manufacturer’s protocol. The efficacy of knockdown was calculated from the data of immunocytochemical staining as follows: the efficacy of knockdown = number of negative cells/number of total cells.

### Membrane preparation and western blot analysis

Plasma membrane fractions were prepared as described previously [23]. The cell pellet was suspended in homogenization buffer containing 10 mM Tris–HCl (pH 7.5), 250 mM sucrose, 100 mM NaCl, 1 mM ethylenediaminetetraacetic acid, and protease inhibitor cocktail (Roche Applied Science, Indianapolis, IN). The cells were homogenized and centrifuged at 1,000 × *g* for 5 min at 4 °C. The supernatant was centrifuged at 430,000 × *g* for 15 min, and the membrane pellet was resuspended in 0.75 mL of 30% iodixanol solution (20 mM Tris–HCl (pH 7.5), 1 mM ethylenediaminetetraacetic acid, 30% (w/v) iodixanol (Axis-Shield PoC AS, Oslo, Norway), and 125 mM sucrose). The membrane suspension was overlaid sequentially by 4 mL each of 25%, 17.5%, 10%, and 2.5% iodixanol solutions, then centrifuged at 100,000 × *g* for 16 h. Each fraction was collected from the top. All fractions were analyzed by sodium dodecyl sulfate–polyacrylamide gel electrophoresis (SDS-PAGE) and western blotting. After confirmation by western blotting with plasma membrane markers, fractions from the 2.5% to 10% iodixanol interface were pooled as the plasma membrane fraction. The protein concentration of each sample was determined using the bicinchoninic acid (BCA) method. The membrane fractions were dissolved in 1% Fos-Choline-12 (Anatrace, Maumee, OH) mixed with Laemmli sample buffer and subjected to SDS-PAGE.

The protein sample was separated by SDS-PAGE using a 10% to 20% gradient polyacrylamide gel, and the separated proteins were transferred electrophoretically to a Hybond-P polyvinylidene difluoride transfer membrane (GE Healthcare, Chicago, IL). The membrane was pre-blocked in Bullet Blocking One blocking solution (Nacalai Tesque, Inc.) at room temperature for 1 h. The membrane was then incubated with the blocking solution containing a 1:10,000 dilution of rabbit anti-human LAT1 polyclonal antibody (TransGenic Inc., Fukuoka, Japan), 1:2,000 dilution of rabbit anti-human ATB^0,+^ polyclonal antibody (Medical & Biological Laboratories Co., Ltd., Nagoya, Japan), 1:1,000 dilution of rabbit anti-human ASCT2 polyclonal antibody (Cell Signaling Technology, Inc., Danvers, MA), 1:100 dilution of rabbit anti-mouse xCT polyclonal antibody (TransGenic Inc.), 1:1,000 dilution of rabbit anti-mouse CD98 polyclonal antibody (Sino Biological Inc., Beijing, China), or 1:100,000 dilution of rabbit anti-human sodium potassium ATPase monoclonal antibody (Abcam Inc., Cambridge, UK). The membrane was treated with a 1:5,000 dilution of horseradish peroxidase-conjugated anti-rabbit immunoglobulin G (IgG; Sigma-Aldrich Co., LLC, St. Louis, MO) and developed using ECL Select Western Blotting Detection Reagent (GE Healthcare) before visualization under an LAS-3000 luminescent image analyzer (Fujifilm Corporation, Tokyo, Japan).

### Immunofluorescent analysis

Cells grown on 8-well chamber slides (Matsunami Glass Ind., Ltd., Osaka, Japan) were briefly washed with Dulbecco’s phosphate-buffered saline (PBS) and fixed with 4% paraformaldehyde for 15 min at room temperature. After washing once with PBS for 5 min, the cells were treated with 0.1% Triton X-100 in PBS for 10 min at room temperature for permeabilization. The cells were washed once with PBS for 5 min, followed by blocking for 60 min at room temperature in PBS containing 4% FBS for LAT1 and ATB^0,+^, PBS containing 5% normal goat serum (NGS) and 0.3% Triton X-100 for ASCT2, and PBS containing 1% NGS for xCT. The cells were then incubated with primary antibodies diluted in PBS containing 4% FBS for LAT1 and ATB^0,+^, PBS containing 1% bovine serum albumin and 0.3% Triton X-100 for ASCT2, and PBS containing 1% NGS for xCT. Rabbit anti-human LAT1 polyclonal antibody (Sigma-Aldrich Co., LLC) was diluted at 1:50, rabbit anti-human ATB^0,+^ polyclonal antibody (Sigma-Aldrich Co., LLC) was diluted at 1:50, rabbit anti-human ASCT2 polyclonal antibody (Cell Signaling Technology, Inc.) was diluted at 1:50, and rat anti-human xCT monoclonal antibody (Cosmo Bio Co., Ltd., Tokyo, Japan) was diluted at 1:100. After an overnight incubation at 4 °C, the cells were washed with PBS, then incubated with Alexa488-conjugated goat anti-rabbit IgG (1:500, Thermo Fisher Scientific) in PBS containing 4% FBS and Hoechest33258 (1:1000, Dojindo Laboratories, Kumamoto, Japan) for 90 min at room temperature for LAT1 and ATB^0,+^, Alexa488-conjugated goat anti-rabbit IgG (1:500, Thermo Fisher Scientific) in PBS containing 1% bovine serum albumin, 0.3% Triton X-100, and Hoechest33258 (1:1000, Dojindo Laboratories) for 120 min at room temperature for ASCT2, and Alexa488-conjugated donkey anti-rat IgG (1:2000, Thermo Fisher Scientific) in PBS containing 4% FBS and Hoechest33258 (1:1000, Dojindo Laboratories) for 60 min at room temperature for xCT. The cells were washed with PBS, then mounted on slides with PBS containing 0.1 M dithiothreitol and 50% glycerol. Immunofluorescent staining was observed under the C1 confocal microscope system (Nikon Instech Co., Ltd., Tokyo, Japan).

### Ligand uptake assay

A ^14^C-labeled ligand uptake assay was performed using each of the overexpressing and mock cell lines grown to 90% to 100% confluence on collagen-coated 24-well plates. The combinations of ligand and inhibitor used were: L-^14^C-leucine (Moravek, Inc., Brea, CA) and 2-amino-2-norbornanecarboxylic acid (BCH; Sigma-Aldrich Co., LLC) for LAT1; ^14^C-glycine (Moravek, Inc.) and α-methyl-DL-tryptophan (AMT; Sigma-Aldrich Co., LLC) for ATB^0,+^; L-^14^C-glutamine (PerkinElmer, Waltham, MA) and L-γ-glutamyl-p-nitroanilide (GPNA; Fujifilm Wako Pure Chemical Corporation) for ASCT2; and L-^14^C-glutamic acid (Moravek, Inc.) and sulfasalazine (SF; Fujifilm Wako Pure Chemical Corporation) for xCT. The incubation and washing solutions used were Na-free Hank’s Balanced Salt Solution (pH 7.4) for LAT1 and xCT, and Hank’s Balanced Salt Solution (pH 7.4) for ATB^0,+^ and ASCT2. First, the cells were washed three times with wash solution, then incubated in the same solution for 10 min at 37 °C. Next, the ^14^C-labeled ligands (1 μM) were added, then incubated for 3 min at 37 °C with or without the corresponding inhibitor (1 mM). The reaction was stopped by washing the cells three times with ice-cold wash solution. The cells were then lysed in 500 μL of 0.1 N NaOH, followed by incubation for 15 min at room temperature. The lysates of each well were collected in scintillation vials and mixed with Clear-sol Ι (Nacalai Tesque, Inc.). The protein concentration of a portion of the lysates was determined using the BCA method. The radioactivity was measured by a Tri-Carb 2800TR scintillation counter (PerkinElmer, Waltham, MA).

An ^18^F-FIMP uptake assay was also performed using each of the overexpressing and mock cell lines grown to 90% to 100% confluence on collagen-coated 12-well plates. The inhibitors and solutions used were the same as described for the ^14^C-labeled ligand uptake assay. First, the cells were washed three times with wash solution, then incubated in the same solution for 10 min at 37 °C. Next, ^18^F-FIMP (370 kBq) was added, then the cells were incubated for 3 min at 37 °C with or without the corresponding inhibitor (1 mM). The reaction was stopped by washing the cells three times with ice-cold wash solution. The cells were then lysed in 1 mL of 0.1 N NaOH, followed by incubation for 15 min at room temperature. The lysates of each well were collected in microtubes. The protein concentration of a portion of the lysates was determined using the BCA method. The radioactivity was measured by a 2480 WIZARD^2^ Auto Gamma Counter (PerkinElmer).

### Probe synthesis

**Fig. 1:**
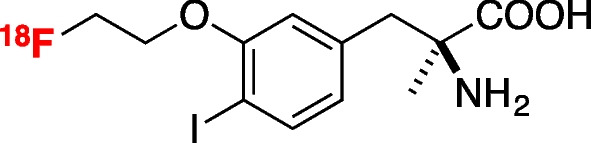
Chemical structure of (*S*)-2-amino-3-[3-(2-^18^F-fluoroethoxy)-4-iodophenyl]-2-methylpropanoic acid (^18^F-FIMP)

^18^F-FIMP (Fig. 1) was synthesized as described previously [20]. Radiochemical purities of > 99.5% were determined by high-performance liquid chromatography.

### Statistical analysis

Data are presented as the mean ± standard deviation. All statistical analyses were performed using Student’s t test on Microsoft Excel 2010 version 14.0 (Microsoft, Redmond, WA). P-values less than 0.05 were considered to be significant.

## Results

### Evaluation of protein expression in transporter-overexpressing cell lines

**Fig. 2:**
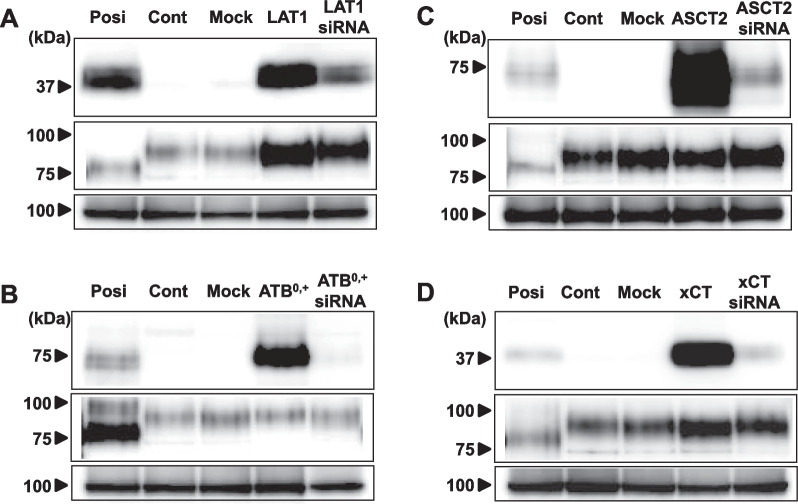
Overexpression of human amino acid transporters. Western blot analyses were performed using anti-human **A** LAT1, **B** ATB^0,+^, **C** ASCT2, and **D** xCT antibodies on membrane protein extracts from the positive-control cells, negative-control cells (CHO-K1 cells), control vector-transfected cells (Mock), expression vector-transfected cells, and gene-specific siRNA-transfected cells (upper panel). The positive-control cells were **A** MCF-7, **B** T3M-4, **C** NCI-H460, and **D** A549 cells. The same blots were also probed with anti-CD98 antibody (middle panel), and anti-Na + /K + -ATPase antibody as a loading control (lower panel).

The protein expression levels in the membrane protein extracts from the positive-control cells, negative-control cells (CHO-K1 cells), control vector-transfected cells (mock), expression vector-transfected cells, and gene-specific siRNA-transfected cells were evaluated by western blot analysis using anti-human LAT1 (Fig. 2A, Suppl. Fig. 1), ATB^0,+^ (Fig. 2B, Suppl. Fig. 2), ASCT2 (Fig. 2C, Suppl. Fig. 3), and xCT (Fig. 2D, Suppl. Fig. 4) antibodies. As positive-control cells, MCF-7 (Fig. 2A, Suppl. Fig. 1), T3M-4 (Fig. 2B, Suppl. Fig. 2), NCI-H460 (Fig. 2C, Suppl. Fig. 3), and A549 (Fig. 2D, Suppl. Fig. 4) cells were used. No signals were observed for the control and mock cells on all blots, whereas intense signals were observed for all of the expression vector-transfected cells. These signals were weak, or strongly reduced, by gene-specific siRNA treatment. The signals of the anti-Na + /K + -ATPase antibody, which was used as a loading control, were similar in intensity to the other signals on the same membrane. Full-length blots images are included in an Additional file 1.

**Fig. 3:**
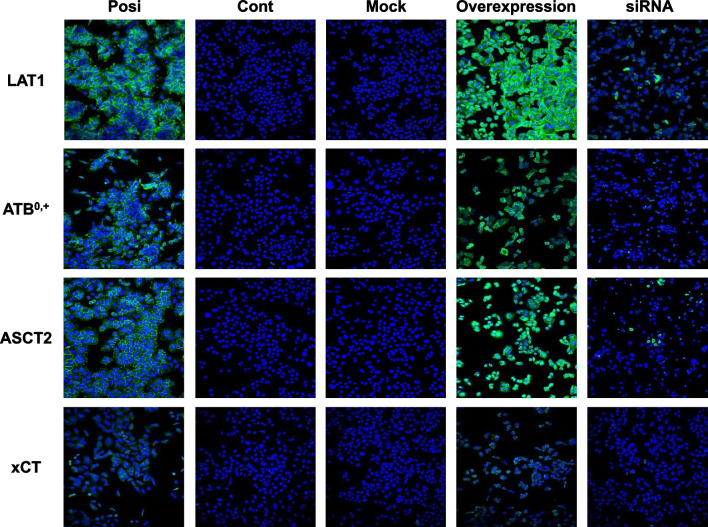
Immunofluorescent analysis of human amino acid transporters. Immunofluorescent analyses were performed using anti-human **A** LAT1, **B** ATB^0,+^, **C** ASCT2, and **D** xCT antibodies (green) on the positive-control cells, negative-control cells (CHO-K1 cells), control vector-transfected cells (Mock), expression vector-transfected cells, and gene-specific siRNA-transfected cells. The positive-control cells were **A** MCF-7, **B** T3M-4, **C** NCI-H460, and **D** A549 cells. Nuclei were visualized by 4',6-diamidino-2-phenylindole (DAPI) staining (blue)

The protein expression levels in transfected cells were also evaluated by immunofluorescent analysis using the same specific antibodies used in the western blot analysis (Fig. 3). No signals were observed for the control and mock cells, whereas intense signals were observed for the expression vector-transfected cells. These signals were strongly reduced by gene-specific siRNA treatment (Table 2).

**Table 2:** Knockdown efficiency at the protein level

	LAT1	ATB^0,+^	ASCT2	xCT
Transfected cells	0	0	0	0.09
	(546/546)	(214/214)	(258/258)	(198/217)
Transfected cells + siRNA	0.92	0.99	0.93	0.98
	(91/1105)	(5/1284)	(77/1156)	(21/1353)

Knockdown efficiency = 1−[(No. of immuno-positive cells)/(No. of total cells)]

siRNA, small interfering ribonucleic acid; No., number; LAT1, L-type amino acid transporter 1; ATB^0,+^, sodium- and chloride-dependent neutral and basic amino acid transporter B(0 +); ASCT2, alanine serine cysteine transporter 2; xCT, cystine/glutamate transporter

### Functional analysis of the overexpressing cell lines

**Fig. 4:**
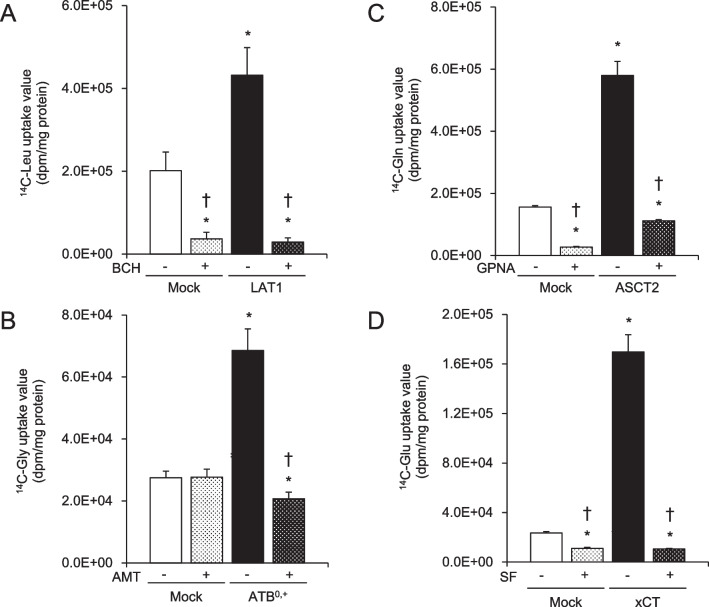
Functional analysis of amino acid transporter-overexpressing cell lines. Transporter-mediated ^14^C-labeled substrate uptake (1 µM) was measured in the presence ( +) or absence (−) of specific inhibitors (BCH, AMT, GPNA, or SF) in control vector-transfected cells (Mock) and expression vector-transfected cells. Data are presented as the mean ± standard deviation (*n* = 4–6). *P < 0.01 when compared to Mock cells without an inhibitor; †*P* < 0.01 when compared to the corresponding overexpressing cells without an inhibitor. AMT, α-Me-DL-Trp; BCH, 2-aminobicyclo-(2,2,1)-heptane-2-carboxylic acid; GPNA, L-γ-glutamyl-p-nitroanilide; SF, sulfasalazine

To evaluate the transport activity of the overexpressing cell lines, transporter-mediated ^14^C-labeled substrate uptake was measured in the presence or absence of specific inhibitors, i.e., BCH, AMT, GPNA, or SF, in the control vector (mock)-transfected cells and each of the expression vector-transfected cell lines (Fig. 4). The uptake values for all of the ^14^C-labeled substrates were significantly higher in the expression vector-transfected cells than in the control (mock) cells. These uptake values were significantly inhibited by each of the corresponding specific inhibitors. In the presence of the inhibitors, the uptake levels were significantly lower in the expression vector-transfected cells than in the control (mock) cells. The uptake values in the control (mock) cells for LAT1, ASCT2, and xCT were significantly inhibited by the corresponding specific inhibitors.

**Fig. 5:**
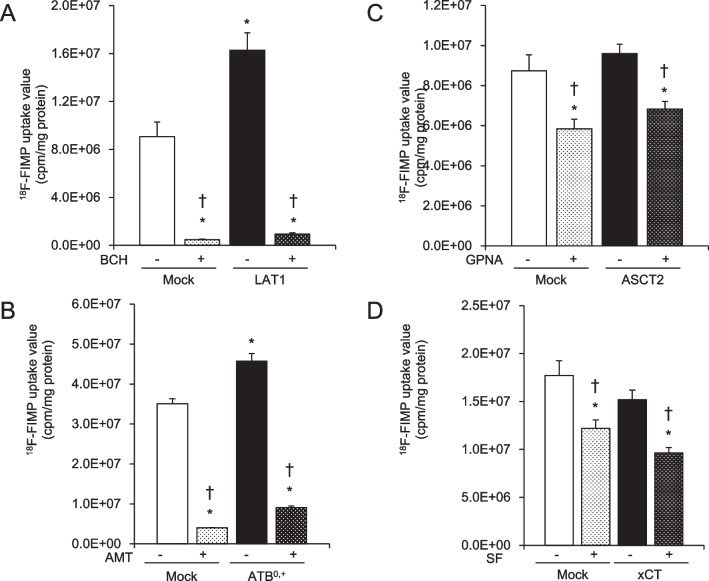
Functional analysis of amino acid transporter-overexpressing cell lines. Transporter-mediated ^18^F-FIMP uptake was measured in the presence ( +) or absence (−) of specific inhibitors (BCH, AMT, GPNA, or SF) in control vector-transfected cells (Mock) and expression vector-transfected cells. Data are presented as the mean ± SD (n = 4–6). *P < 0.01 when compared to mock cells without an inhibitor; †*P* < 0.01 when compared to the corresponding overexpressing cells without an inhibitor. AMT, α-Me-DL-Trp; BCH, 2-aminobicyclo-(2,2,1)-heptane-2-carboxylic acid; GPNA, L-γ-glutamyl-p-nitroanilide; SF, sulfasalazine.

To estimate the affinity of ^18^F-FIMP in each transporter-overexpressing cell line, transporter-mediated ^18^F-FIMP uptake was measured in the presence or absence of specific inhibitors in the control vector (mock)-transfected cells and each of the expression vector-transfected cell lines (Fig. 5). ^18^F-FIMP uptake values were significantly higher in the LAT1- and ATB^0,+^-overexpressing cell lines than in the control (mock) cells. These uptake values were significantly decreased by the specific inhibitors BCH and AMT (Fig. 5A and B). In contrast, the ^18^F-FIMP uptake values in the ASCT2- and xCT-overexpressing cell lines were not significantly higher than those in the control (mock) cells (Fig. 5C and D). The ^18^F-FIMP uptake values in the control (mock) cells were significantly inhibited by each of the specific inhibitors, i.e., BCH, AMT, GPNA, and SF.

## Discussion

We previously reported that FIMP has high affinity for LAT1 and low affinity for LAT2 [20]. FIMP has a similar chemical structure as tyrosine, which is a substrate not only for LAT1, but also for ATB^0,+^ [11]. Therefore, we hypothesized that FIMP might have affinity not only for LAT1, but also for ATB^0,+^. To test this hypothesis, we established cells that overexpressed amino acid transporters LAT1, ATB^0,+^, ASCT2, or xCT. These cells were confirmed by western blot (Fig. 2) and immunocytofluorescent (Fig. 3) analyses to have intense protein expression levels for each of the amino acid transporters. LAT1 and xCT forms a heterodimeric amino acid transporter that interacts with the glycoprotein CD98 (SLC3A2; 4F2hc) through a conserved disulfide bond. These transporters cannot perform their functions alone, but can exert their functions only by forming heterodimers with CD98 (4F2hc) protein [7, 24, 25]. Therefore, we also confirmed the protein expression levels of CD98 in these cells by western blot analysis. Intense or weak CD98 signals were observed for the LAT1 (Fig. 2A) and xCT (Fig. 2D) expression vector-transfected cells. These results indicated that the cells overexpressing LAT1 and xCT might have normal transport function. In fact, all transfected cells showed significantly higher substrate uptake levels when compared to the mock cells, and the uptake levels were suppressed by each specific inhibitor (Fig. 4). Furthermore, in support of our hypothesis, our study revealed that FIMP had affinity not only for LAT1, but also for ATB^0,+^ (Fig. 5).

The LAT1-specific inhibitors JPH203 [26] and SKN103 [27] are currently under development as therapeutic agents, and ^18^F-FAMT is being considered as a PET probe to be the companion diagnostic agent [28]. Since ^18^F-FIMP is not a LAT1-specific PET probe, it may be unsuitable as a companion diagnostic agent for LAT1-specific inhibitors. However, ^18^F-FIMP may be more sensitive for tumor detection than LAT1-specific probes. Since multiple amino acid transporters are targeted, the amount of probe accumulation in tumor tissues would be greater than when a single amino acid transporter is targeted. However, to prove this hypothesis, it is necessary to perform further studies that compare ^18^F-FAMT and ^18^F-FIMP in the future.

Only the affinity between ^18^F-FIMP and four typical transporters related to cancer was examined in the present study. However, because there are many other transporters expressed in normal cells and tumor cells, the detailed mechanism of ^18^F-FIMP accumulation in tumors and other tissues remains to be clarified. Especially, SLC38A1 (SNAT1, Sodium-coupled Neutral Amino Acid Transporter 1, Solute Carrier Family 38 Member 1) is also highly involved in tumor growth, and examining their expression by PET imaging is critical for diagnosis and PET imaging of their expression is very important for diagnosis and treatment options [29, 30]. It will be necessary to evaluate the transport function of ^14^C-AIB and NMe AIB by cellular uptake assay using ^18^F-FIMP in the future.

## Conclusions

We demonstrated that ^18^F-FIMP has affinity not only for LAT1, but also for ATB^0,+^. These results may be useful for understanding the mechanisms of the whole-body distribution and tumor accumulation of ^18^F-FIMP. And, as the specificity of ^18^F-FIMP for amino acid transporters is elucidated, this PET imaging technique may provide important information for cancer diagnosis and treatment selection.

## Supplementary Information

**Additional file 1**. Full-length western blots images.

## Data Availability

The datasets used and/or analyzed during the current study are available from the corresponding author on reasonable request.
