# Supplementary material for: In vitro evaluation of (S)-2-amino-3-[3-(2-18F-fluoroethoxy)-4-iodophenyl]-2-methylpropanoic acid (18F-FIMP) as a positron emission tomography probe for imaging amino acid transporters
Source: EJNMMI Res. 2023 Apr 28;13:36. doi: 10.1186/s13550-023-00988-1 (PMC10147893; doi:10.1186/s13550-023-00988-1)
Supplement: Supplementary file 1 — Additional file 1. Full-length western blots images. [file 13550_2023_988_MOESM1_ESM.pptx]

## Slide 1
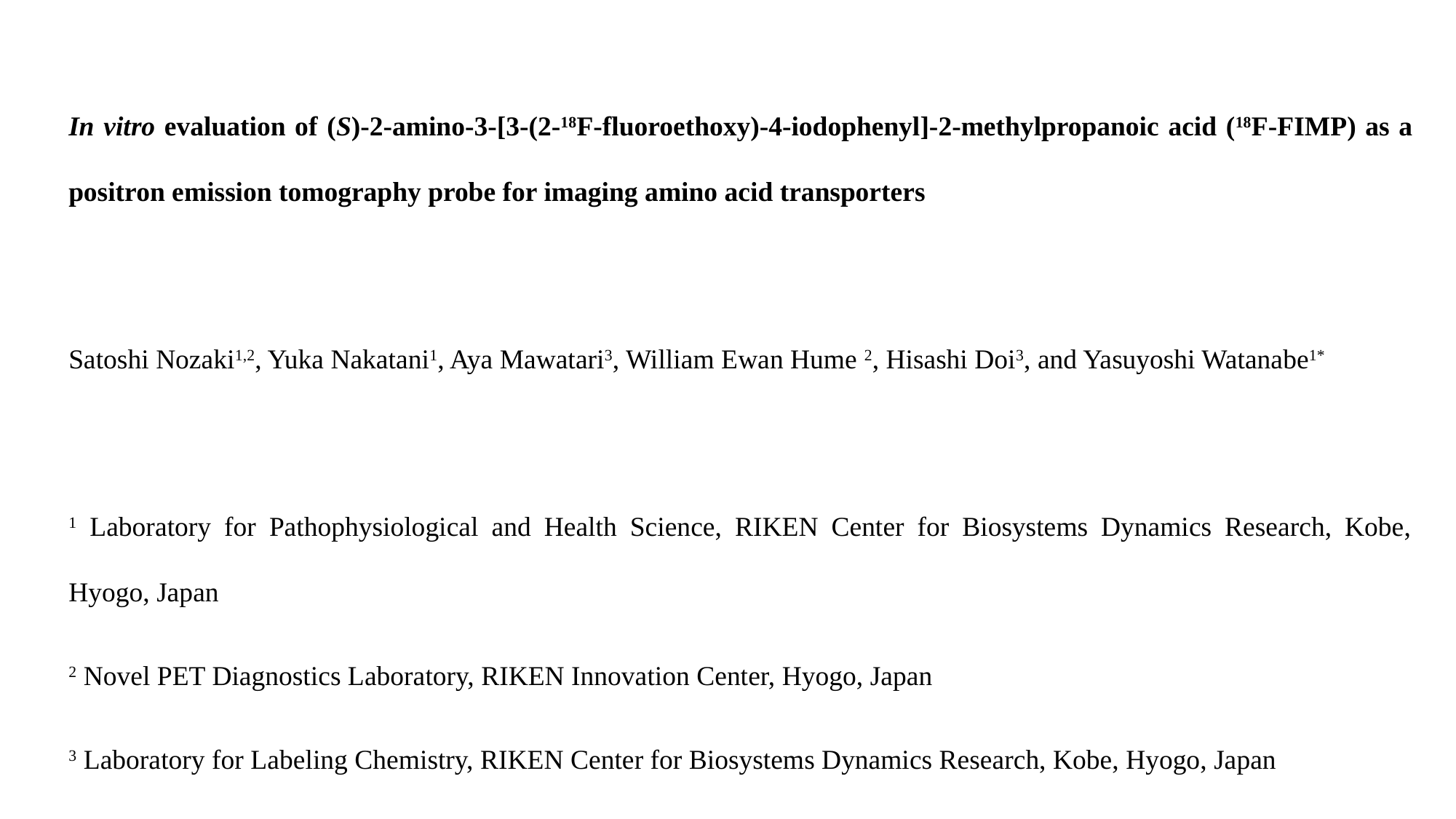

In vitro evaluation of (S)-2-amino-3-[3-(2-18F-fluoroethoxy)-4-iodophenyl]-2-methylpropanoic acid (18F-FIMP) as a positron emission tomography probe for imaging amino acid transporters
Satoshi Nozaki1,2, Yuka Nakatani1, Aya Mawatari3, William Ewan Hume 2, Hisashi Doi3, and Yasuyoshi Watanabe1*
1 Laboratory for Pathophysiological and Health Science, RIKEN Center for Biosystems Dynamics Research, Kobe, Hyogo, Japan
2 Novel PET Diagnostics Laboratory, RIKEN Innovation Center, Hyogo, Japan
3 Laboratory for Labeling Chemistry, RIKEN Center for Biosystems Dynamics Research, Kobe, Hyogo, Japan

## Slide 2
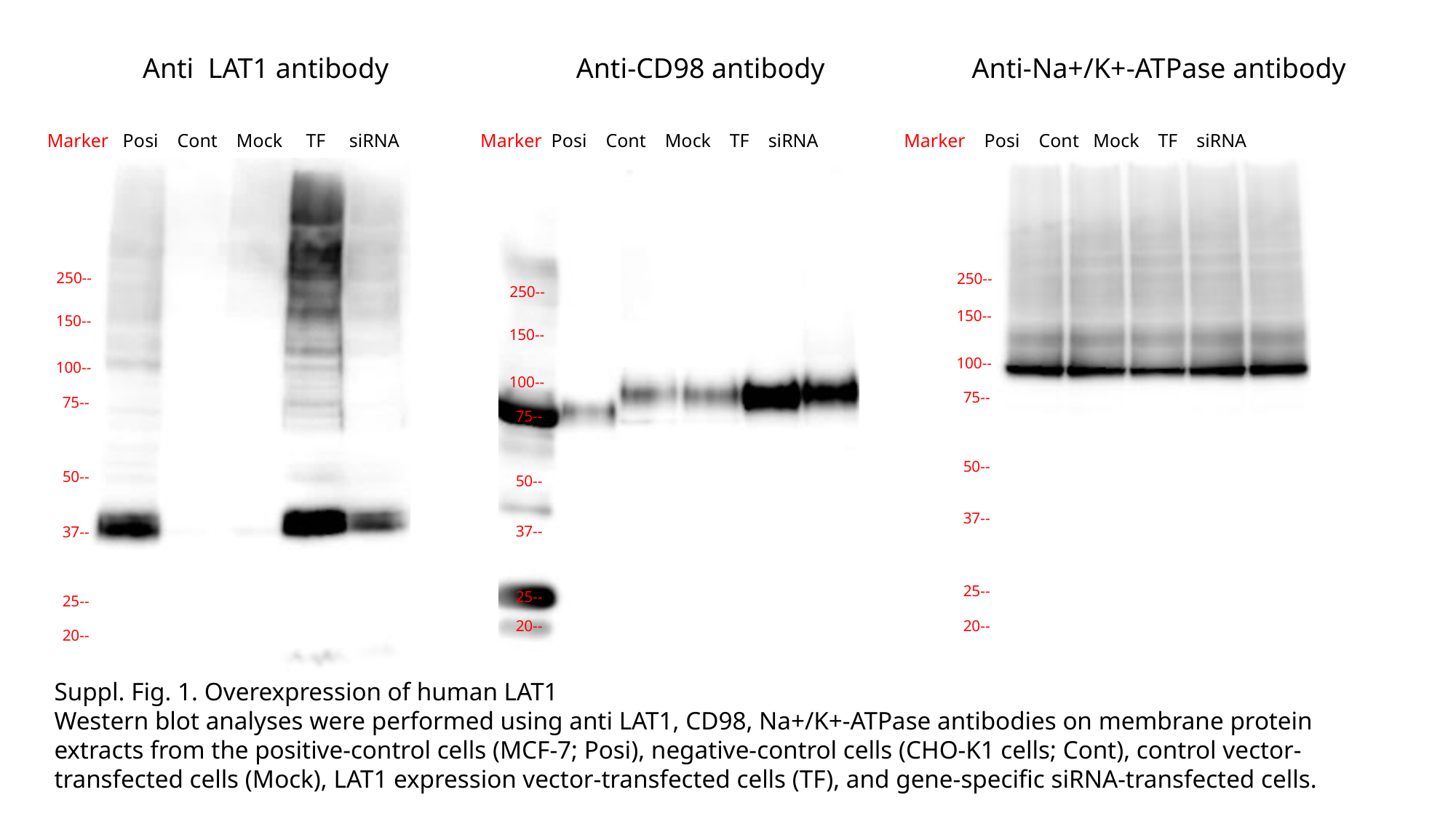

Anti LAT1 antibody
Anti-CD98 antibody
Anti-Na+/K+-ATPase antibody
Marker Posi Cont Mock TF siRNA Marker Posi Cont Mock TF siRNA Marker Posi Cont Mock TF siRNA
250--
150--
100--
75--
50--
37--
25--
20--
250--
150--
100--
75--
50--
37--
25--
20--
250--
150--
100--
75--
50--
37--
25--
20--
Suppl. Fig. 1. Overexpression of human LAT1
Western blot analyses were performed using anti LAT1, CD98, Na+/K+-ATPase antibodies on membrane protein extracts from the positive-control cells (MCF-7; Posi), negative-control cells (CHO-K1 cells; Cont), control vector-transfected cells (Mock), LAT1 expression vector-transfected cells (TF), and gene-specific siRNA-transfected cells.

## Slide 3
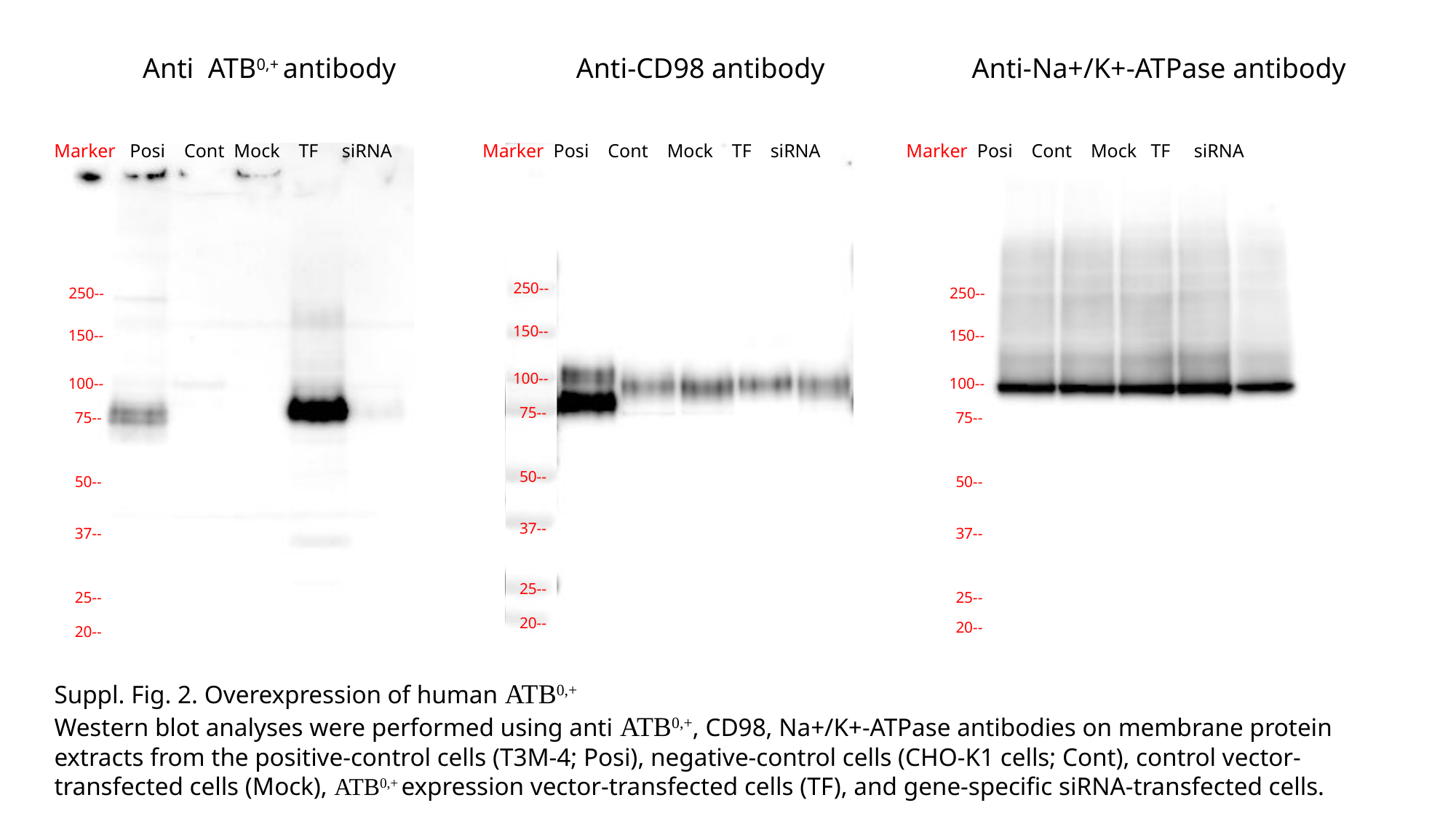

Anti ATB0,+ antibody
Anti-CD98 antibody
Anti-Na+/K+-ATPase antibody
 Marker Posi Cont Mock TF siRNA Marker Posi Cont Mock TF siRNA Marker Posi Cont Mock TF siRNA
250--
150--
100--
75--
50--
37--
25--
20--
250--
150--
100--
75--
50--
37--
25--
20--
250--
150--
100--
75--
50--
37--
25--
20--
Suppl. Fig. 2. Overexpression of human ATB0,+
Western blot analyses were performed using anti ATB0,+, CD98, Na+/K+-ATPase antibodies on membrane protein extracts from the positive-control cells (T3M-4; Posi), negative-control cells (CHO-K1 cells; Cont), control vector-transfected cells (Mock), ATB0,+ expression vector-transfected cells (TF), and gene-specific siRNA-transfected cells.

## Slide 4
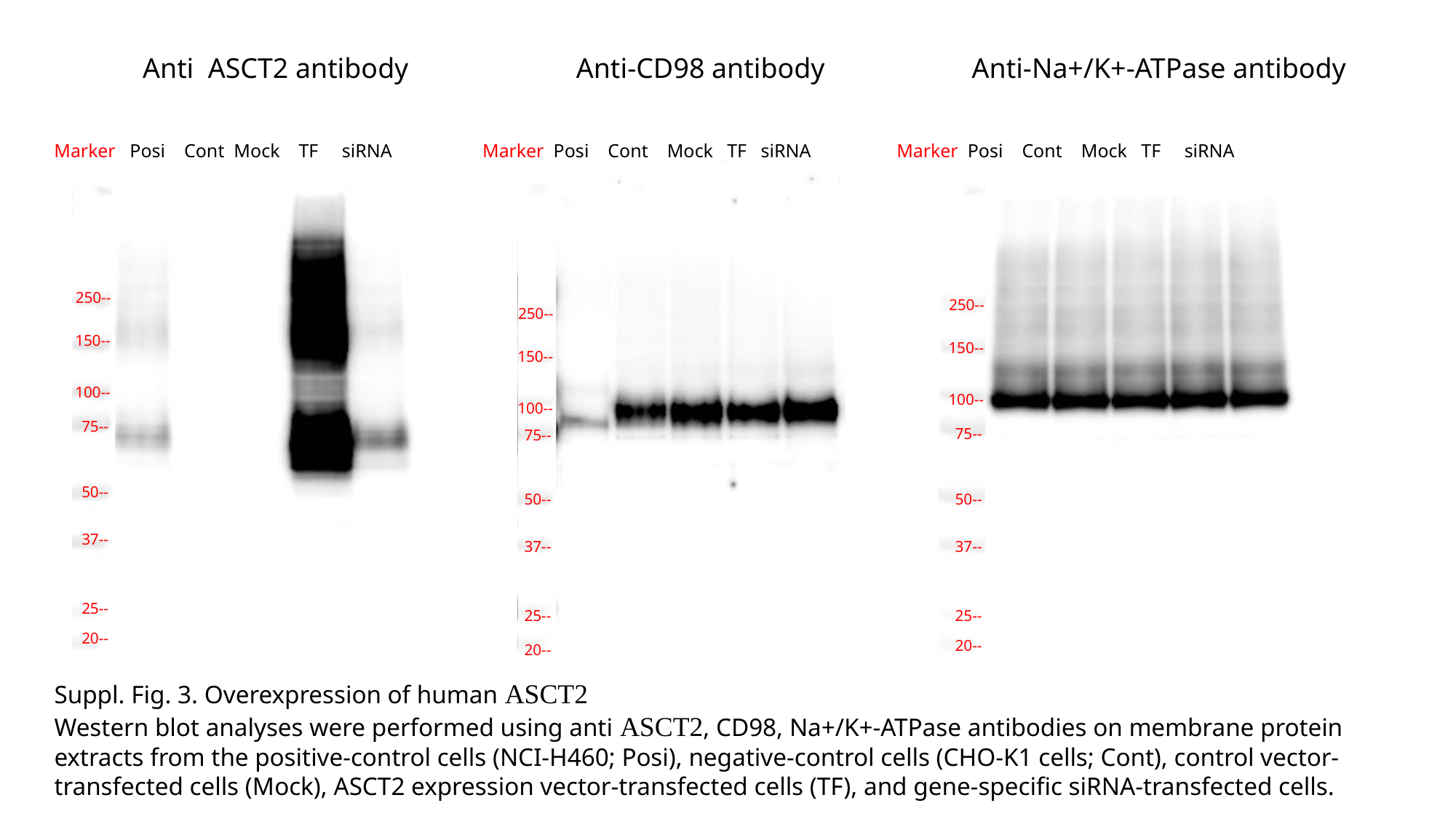

Anti ASCT2 antibody
Anti-CD98 antibody
Anti-Na+/K+-ATPase antibody
 Marker Posi Cont Mock TF siRNA Marker Posi Cont Mock TF siRNA Marker Posi Cont Mock TF siRNA
250--
150--
100--
75--
50--
37--
25--
20--
250--
150--
100--
75--
50--
37--
25--
20--
250--
150--
100--
75--
50--
37--
25--
20--
Suppl. Fig. 3. Overexpression of human ASCT2
Western blot analyses were performed using anti ASCT2, CD98, Na+/K+-ATPase antibodies on membrane protein extracts from the positive-control cells (NCI-H460; Posi), negative-control cells (CHO-K1 cells; Cont), control vector-transfected cells (Mock), ASCT2 expression vector-transfected cells (TF), and gene-specific siRNA-transfected cells.

## Slide 5
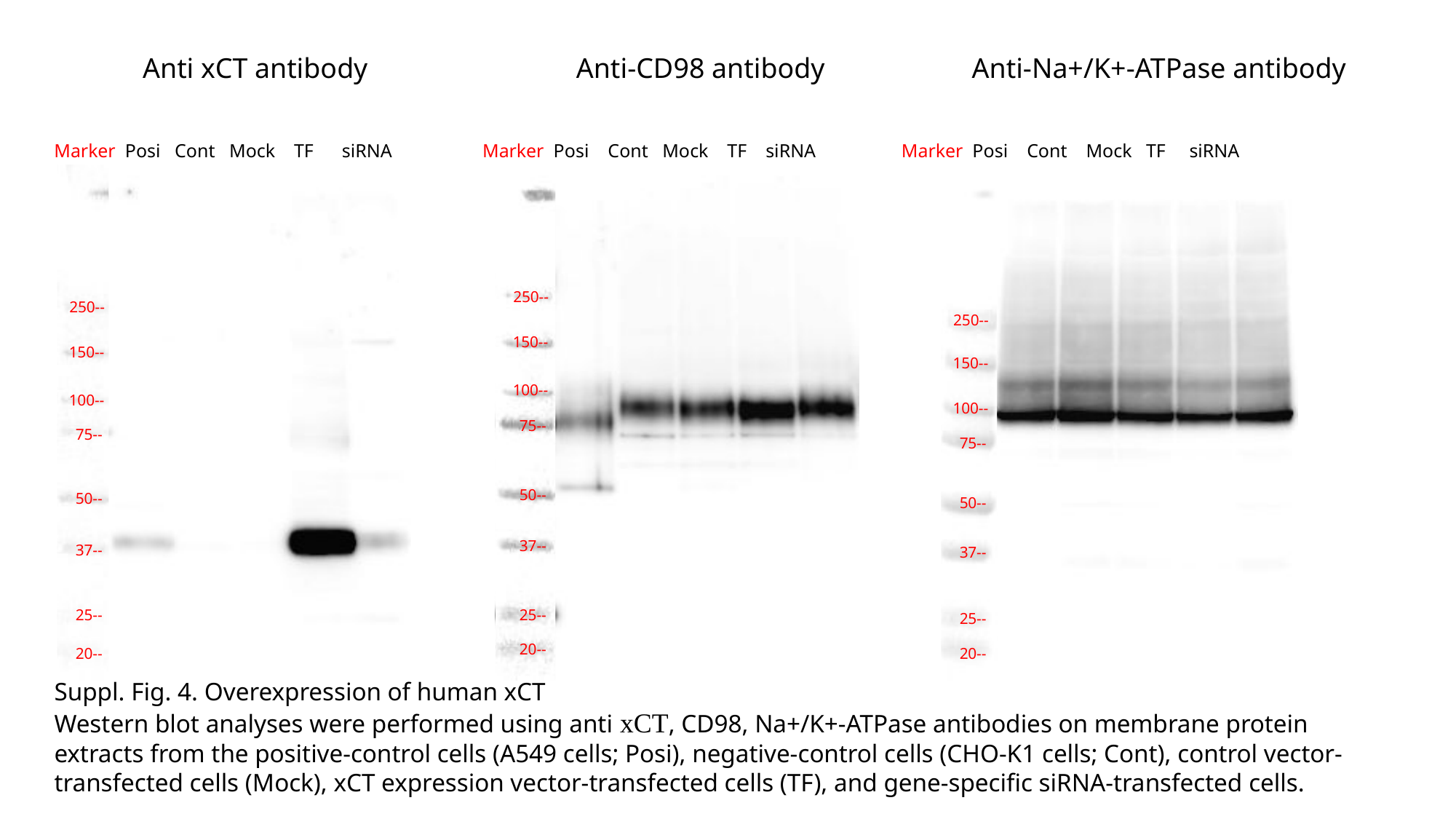

Anti xCT antibody
Anti-CD98 antibody
Anti-Na+/K+-ATPase antibody
 Marker Posi Cont Mock TF siRNA Marker Posi Cont Mock TF siRNA Marker Posi Cont Mock TF siRNA
250--
150--
100--
75--
50--
37--
25--
20--
250--
150--
100--
75--
50--
37--
25--
20--
250--
150--
100--
75--
50--
37--
25--
20--
Suppl. Fig. 4. Overexpression of human xCT
Western blot analyses were performed using anti xCT, CD98, Na+/K+-ATPase antibodies on membrane protein extracts from the positive-control cells (A549 cells; Posi), negative-control cells (CHO-K1 cells; Cont), control vector-transfected cells (Mock), xCT expression vector-transfected cells (TF), and gene-specific siRNA-transfected cells.
